# Markers of Disease Severity Are Associated with Malnutrition in Parkinson's Disease

**DOI:** 10.1371/journal.pone.0057986

**Published:** 2013-03-27

**Authors:** Jamie M. Sheard, Susan Ash, George D. Mellick, Peter A. Silburn, Graham K. Kerr

**Affiliations:** 1 School of Exercise and Nutrition Sciences, Queensland University of Technology, Brisbane, Queensland, Australia; 2 Movement Neuroscience Program, Institute of Health and Biomedical Innovation, Queensland University of Technology, Brisbane, Queensland, Australia; 3 Australia Eskitis Institute for Cell and Molecular Therapies, Griffith University, Brisbane, Queensland, Australia; 4 University of Queensland Centre for Clinical Research, Brisbane, Queensland, Australia; Oslo University Hospital, Norway

## Abstract

**Objective:**

In Parkinson's disease (PD), commonly reported risk factors for malnutrition in other populations commonly occur. Few studies have explored which of these factors are of particular importance in malnutrition in PD. The aim was to identify the determinants of nutritional status in people with Parkinson's disease (PWP).

**Methods:**

Community-dwelling PWP (>18 years) were recruited (n = 125; 73M/52F; Mdn 70 years). Self-report assessments included Beck's Depression Inventory (BDI), Spielberger Trait Anxiety Inventory (STAI), Scales for Outcomes in Parkinson's disease – Autonomic (SCOPA-AUT), Modified Constipation Assessment Scale (MCAS) and Freezing of Gait Questionnaire (FOG-Q). Information about age, PD duration, medications, co-morbid conditions and living situation was obtained. Addenbrooke's Cognitive Examination (ACE-R), Unified Parkinson's Disease Rating Scale (UPDRS) II and UPDRS III were performed. Nutritional status was assessed using the Subjective Global Assessment (SGA) as part of the scored Patient-Generated Subjective Global Assessment (PG-SGA).

**Results:**

Nineteen (15%) were malnourished (SGA-B). Median PG-SGA score was 3. More of the malnourished were elderly (84% vs. 71%) and had more severe disease (H&Y: 21% vs. 5%). UPDRS II and UPDRS III scores and levodopa equivalent daily dose (LEDD)/body weight(mg/kg) were significantly higher in the malnourished (Mdn 18 vs. 15; 20 vs. 15; 10.1 vs. 7.6 respectively). Regression analyses revealed older age at diagnosis, higher LEDD/body weight (mg/kg), greater UPDRS III score, lower STAI score and higher BDI score as significant predictors of malnutrition (SGA-B). Living alone and higher BDI and UPDRS III scores were significant predictors of a higher log-adjusted PG-SGA score.

**Conclusions:**

In this sample of PWP, the rate of malnutrition was higher than that previously reported in the general community. Nutrition screening should occur regularly in those with more severe disease and depression. Community support should be provided to PWP living alone. Dopaminergic medication should be reviewed with body weight changes.

## Introduction

Well-documented risk factors for poor nutrition in community-dwelling adults include older age [1], [2], living alone [3], dementia [4], [5], depression [1], [3], [5], anorexia [6], gastrointestinal dysfunction (dysphagia, slow gastric emptying, constipation) [7], [8], poor functional status [3], [5], co-morbidities [9] and polypharmacy [3].

In Parkinson's disease (PD), these risk factors are common, often occurring more frequently than in age-matched controls, including dementia [10], depression [11] and gastrointestinal disorders (dysphagia [12], [13], early satiety [12] and constipation [13]). While limited research has been conducted to determine the predictors of malnutrition in PD, it has been reported that depression and constipation are significant predictors of malnutrition [14].

Factors that are specific to PD that may place someone at nutritional risk include the motor symptoms of bradykinesia, akinesia, rigidity, and tremor which can impair functional ability and make it difficult to ambulate [15], shop, cook, and feed independently [16], [17]. Decreased hand-mouth coordination and difficulties completing fine movements, such as that required with utensils [15] can be present.

Decreased weight and body mass indices are not associated with longer disease duration [18]. However, it has been reported that disease severity (Hoehn & Yahr) is associated with decreased body mass indices (BMI) [18], but Hoehn & Yahr classification does not significantly predict a diagnosis of malnutrition using the Mini-Nutritional Assessment (MNA) [14].

The use of levodopa as medical management for PD can introduce side effects such as nausea & vomiting and weight loss [19]. Higher intakes of levodopa have been associated with lower BMIs [20], particularly higher intakes per kilogram of body weight [21]. This could potentially be due to the fact that higher levodopa dosages increase the risk of developing dyskinesias [22] and therefore increase energy expenditure and energy requirements [21].

While many studies have investigated relationships between these potential risk factors and decreased BMIs, few have explored the relationship with nutritional status using a diagnosis of malnutrition based on a nutrition assessment tool. BMI is not sensitive enough on its own to identify malnutrition [23], and nutrition assessment tools incorporate anthropometry, weight history, dietary intake and physical signs of malnutrition to diagnosis malnutrition.

Malnutrition is under-recognised across all healthcare settings [24] but particularly in the community where, firstly, there is limited data on the extent of malnutrition and, secondly, access to people at nutrition risk can be difficult [24], [25]. Poor nutritional status is an independent risk factor for hospitalization [3], and community dwelling adults just transitioning to aged care have poorer nutritional status than other adults in the community [26]. Therefore, identifying and understanding the issue in the community can assist with intervention planning and implementation which may prevent or delay declines in nutritional status and potentially admittance to hospital or aged care [24]. Raising awareness of the issue of malnutrition in people with Parkinson's disease (PWP) in the community, therefore, may assist with advocacy for nutrition screening by health professionals with whom they have regular contact.

Therefore, the aim of the current study was to identify which factors predict nutritional status in PWP free-living in the community when measured by the Subjective Global Assessment (SGA), a valid nutrition assessment tool for use in the community [24] and previously used to assess nutritional status in community-dwelling elderly [25], oncology patients [27], [28], and patients with chronic kidney disease [29], [30].

## Methods

### Recruitment process

Community-dwelling PWP, aged >18 years were recruited between February and August 2011 using a variety of methods including media releases in local newspapers and inclusion in the quarterly newsletter issued by Parkinson's Queensland, Inc, the local non-profit organization [31]. Potential participants initiated contact with the research team to participate. Participants were excluded only if they resided in an aged care/long-term care facility. All other consenting adults >18 years with PD were included. Geographical location was limited to areas within ∼2 hour driving distance from Brisbane, Queensland, Australia.

### Ethics statement

Informed written consent was obtained as per protocol approved by the Queensland University of Technology Human Research Ethics Committee, which also approved the study (#1000001150).

### Setting

Data collection was completed either at Queensland University of Technology (n = 15) or participants' homes (n = 110).

### Demographic information and medical history

Birth date, disease duration since diagnosis (PD duration), medications, co-morbid conditions and living situation were obtained from the participant and/or their spouse. The number of prescription medications and number of comorbid conditions were each split into 2 categories: (<4 medications, ≥4 medications) and (<4 conditions, ≥4 conditions) [5]. Levodopa equivalent daily doses (LEDD) [32] and LEDD per kilogram body weight (mg/kg) (levodopa/body weight) were calculated.

### Cognitive function and Parkinson's disease rating scales

Cognitive function was measured using the Addenbrooke's Cognitive Examination (ACE-R), which includes the Mini-Mental State Examination (MMSE). The Unified Parkinson's Disease Rating Scale (UPDRS) motor sub-scale (III) was conducted following a medication dose, generally approaching optimal state, to assess disease severity. The postural stability component of the assessment was not completed to ensure the safety of the participants and the researcher. The UPDRS Activities of Daily Living sub-scale (UPDRS II) was also completed. The Hoehn and Yahr (H&Y) scale, which is a five-point scale (1–5) with a higher rating on the scale indicating a greater amount of disability and impairment, was also measured. The H&Y scores were also split into 2 groups (less severe PD H&Y 0–3, severe PD H&Y 4–5).

### Anthropometry and nutritional status

Body weight was measured to the nearest 0.1 kg (Tanita HD-316, Japan) in light clothing, without shoes. Nutritional status was measured by a dietitian using the SGA, resulting in a categorisation of nutrition status: SGA-A (well nourished), SGA-B (moderately malnourished) or SGA-C (severely malnourished) [33]. The scored Patient-Generated Subjective Global Assessment (PG-SGA) was also completed, providing a score of the total of four worksheet scores, with a higher score indicating poorer nutritional status [34]. PG-SGA Worksheet 1, completed by the participant, provides a score for recent changes in weight, food intake, nutrition impact symptoms (lack of appetite, nausea/vomiting, changes in smell and taste, constipation, dry mouth, mouth sores, early satiety, pain, difficulties swallowing), and functional capacity. Worksheet 2 provides a score for medical conditions and age. Worksheet 3 provides a score for components of metabolic stress, and finally Worksheet 4 consists of the physical examination score. The assessor completes worksheets 2, 3 and 4.

### Self-completed questionnaires

Participants received the Beck's Depression Inventory (BDI), the Spielberger Trait Anxiety Inventory (STAI), the Scales for Outcomes in Parkinson's disease – Autonomic (SCOPA-AUT), the Modified Constipation Assessment Scale (MCAS) and the Freezing of Gait Questionnaire (FOG-Q) approximately 1 week before their visit. They were asked to complete the questionnaires at home at their convenience. The 6 sub-scores of the SCOPA-AUT were calculated: gastrointestinal, urinary, cardiovascular, thermoregulatory, pupillomotor and sexual.

### Statistical analysis

Variables of interest were not normally distributed. Therefore, non-parametric Mann-Whitney U tests were conducted to compare scores between the groups. Pearson's Chi square tests were used to evaluate the differences in categorical variables except where cell counts were <5 (H&Y, MCAS satisfaction rating).

Univariable logistic regression with SGA category (SGA-A, SGA-B) as the outcome variable was conducted to determine crude unadjusted odds ratios. Multivariable logistic regression analysis was performed to include all variables with an identified association with outcome in the univariable analysis. A final statistical model, including the most significant variables, was constructed using the backward elimination procedure.

We also used regression modeling to examine the determinants of PG-SGA score using the same variables of interest as the logistic regression. The PG-SGA scores were positively skewed, therefore the PG-SGA score was log-transformed to produce a normal distribution. Univariate multifactorial analysis of variance was conducted using backward elimination to remove the most insignificant variables from the statistical model to produce the significant final model for predicting PG-SGA score.

Statistical analysis was completed using SPSS Version 19 (SPSS Inc., Chicago, IL, USA). Statistical significance was set at p<0.05.

## Results

### Sample characteristics

Of the 125 participants (73 M, 52 F), 15% (n = 19) were moderately malnourished (SGA-B), and none were severely malnourished (SGA-C). Median PG-SGA score was 3 (range 0–15). Median age of the participants was 70.0 years (range 35.0–92.0 years), and 73% (n = 91) were aged 65 years or older. Self-reported median length of disease was 6.0 years (range 0.0–31.0 years).

### Missing data

One participant could not recall when diagnosis had occurred. Missing assessments included UPDRS II for one participant, UPDRS III for two participants and ACE-R for three participants. These participants did not wish to complete the full assessment. One participant did not complete any of the self-administered questionnaires (BDI, STAI, SCOPA-AUT, MCAS (including the MCAS satisfaction rating), FOG-Q). One participant completed the MCAS but not the satisfaction rating, and one participant completed all questionnaires but the STAI. Those cases with missing data were included in the analysis, and missing data were treated as missing data when that variable was included in the analysis.

### Age and medical characteristics

Age, age at diagnosis and PD duration were not significantly different between the SGA-A and SGA-B groups. However PG-SGA score was significantly different (Table 1). More of the malnourished group were elderly with 84% aged ≥65 years compared to 71% of the well-nourished. When comparing the categorical variables gender, co-morbid conditions, living situation (alone vs with others) and prescription medications, there were no differences between the groups (Tables 1, 2). The most common medical conditions being actively treated were hypertension (n = 25), history of cardiovascular disease (eg, heart attack, placement of stents, pacemaker) (n = 23), hypercholesterolemia (n = 19), osteoporosis (n = 16) and Type 2 diabetes (n = 9). Other conditions included gastrointestinal dysfunction (total n = 7: n = 3 diverticular disease; n = 1 ulcerative colitis; n = 1 irritable bowel syndrome; n = 1 Barrett's esophagus; n = 1 coeliac disease), hypothyroidism (n = 3) and kidney failure (n = 1). Eleven people had a history of cancer, but none were undergoing treatment at the time of the study. LEDD/body weight (mg/kg) were significantly higher in the malnourished participants, *U* = 1340.0, *z* = 2.29, *p* = .022, while LEDD were not.

**Table 1:** Demographic and motor symptom characteristics of the participants compared between nutritional states.

	SGA Classification	
	A (Well-nourished)	B (Moderately malnourished)
	(n = 106)	(n = 19)
	Median (Range)	Median (Range)
Age (years)	69.0 (35.0–92.0)	74.0 (61.0–87.0)
PG-SGA score	2 (0–15)*	8 (4–15)*
Living Situation (with others)	90 (85%)	15 (79%)
PD Duration (years)	6.0 (0.0–31.0)	7.0 (1.0–26.0)
	(n = 105)	
Age at diagnosis (years)	63.0 (28.0–84.0)	66.0 (43.0–84.0)
	(n = 105)	
LEDD (mg)	574.25 (0.0–2296.0)	600.0 (75.0−1600.0)
LEDD/weight (mg/kg)	7.6 (0.0–27.8)*	10.1(1.6–27.9)*
no. of prescription medications	5 (0–16)	5 (0–12)
no. of comorbid conditions	1 (0–6)	1 (0–3)
H&Y†	*	*
0	2 (2%)	0 (0%)
1	27 (25%)	1 (5%)
2	37 (35%)	7 (37%)
3	35 (33%)	7 (37%)
4	5 (5%)	3 (16%)
5	0 (0%)	1 (5%)
Less severe PD (H&Y 0–3)†	101 (96%)*	15 (79%)*
Severe PD (H&Y 4–5)†	5 (4%)*	4 (21%)*
UPDRS III	15 (1–37)*	20 (11–39)*
		(n = 17)
UPDRS II	13 (2–35)*	18 (7–38)*
FOG-Q	7 (0–23)	7 (0–17)

† Reported as frequencies (percentages).

* Statistically significant difference between SGA classifications (p<0.05).

Abbreviations: H&Y = Hoehn & Yahr; FOG-Q = Freezing of Gait Questionnaire; LEDD = levodopa equivalent daily doses; PD = Parkinson's disease; PG-SGA = Patient Generated Subjective Global Assessment; SGA = Subjective Global Assessment; UPDRS = Unified Parkinson's Disease Rating Scale.

**Table 2:** Non-motor symptoms of the participants compared between nutritional states.

	SGA Classification	
	A (Well-nourished)	B (Moderately malnourished)
	(n = 106)	(n = 19)
	Median (Range)	Median (Range)
ACE-R	88 (55–100)	84 (64–95)
	(n = 105)	(n = 17)
MMSE	29 (22–30)*	27 (24–30)*
	(n = 105)	(n = 17)
BDI	8 (0–34)*	14 (5–29)*
	(n = 105)	
STAI	37.5 (20–63)	36 (25–60)
	(n = 105)	(n = 18)
MCAS Satisfaction	4 (1–5)	3 (1–5)
	(n = 104)	
MCAS	3 (0–10)	5 (0–8)
	(n = 105)	
SCOPA-AUT	15 (3–50)	18 (3–33)
	(n = 105)	
SCOPA-AUT gastrointestinal sub-score	4 (0–16)*	6 (0–15)*
	(n = 105)	
SCOPA-AUT urinary sub-score	5 (0–18)	7 (2–11)
	(n = 105)	
SCOPA-AUT cardiovascular sub-score	1 (0–6)	0 (0–4)
	(n = 105)	
SCOPA-AUT thermoregulatory sub-score	2 (0–9)	3 (0–6)
	(n = 105)	
SCOPA-AUT pupillomotor sub-score	1 (0–3)	0 (0–2)
	(n = 105)	
SCOPA-AUT sexual sub-score	0 (0–6)	1 (0–6)
	(n = 105)	

* Statistically significant difference between SGA classifications (p<0.05).

Abbreviations: ACE-R = Addenbrooke's Cognitive Examination-Revised; BDI = Beck's Depression Inventory; MCAS = Modified Constipation Assessment Scale; MMSE = Mini-Mental State Examination; SCOPA-AUT = Scales for Outcomes in Parkinson's disease – Autonomic; SGA = Subjective Global Assessment; STAI = Spielberger Trait Anxiety Inventory.

### Cognition and Parkinson's disease rating scales

The malnourished participants scored significantly lower on the MMSE, *U* = 551.5, *z = *−2.61, *p* = .009, as well as on the visuospatial, *U* = 576.5, *z = *−2.48, *p* = .013, and attention and orientation, *U* = 570.0, *z* = −3.03, *p* = .002 ACE-R sub-scores but not on the total ACE-R score (Table 2). Both the UPDRS II and UPDRS III scores were significantly higher in the malnourished participants (*U* = 1259.5, *z = *2.63, *p* = .009; *U* = 1376.5, *z* = 2.63, *p* = .008, respectively). H&Y categorisation (Table 1) was also significantly different between the groups (Fishers Exact Test p = 0.03). Severe PD (ie H&Y 4 or 5) was significantly more common in the malnourished group (OR = 5.39 (95%CI = 1.06–27.05).

### Non-motor symptoms

The malnourished participants scored significantly higher on the BDI (more depressive symptoms) than the well-nourished participants, *U* = 1457.5, *z* = 3.20, *p* = .001. The total SCOPA-AUT score was not significantly different between the groups, but the gastrointestinal sub-score was significantly higher (more severe gastrointestinal symptoms) in the malnourished participants, *U* = 1283.5, *z = *2.00, *p* = .046.

### Regression analysis

Unadjusted univariable logistic regression analysis revealed relationships between several variables and SGA-B (Table 3). The final multivariable logistic regression model, which included all significantly associated variables using a backwards elimination procedure, identified age at diagnosis, LEDD/body weight and scores for UPDRS III, STAI and BDI as the most significant predictors of malnutrition (SGA-B) (Table 3).

**Table 3:** Significant predictors of malnutrition (SGA-B) and corresponding odds ratios.

	Univariable model Unadjusted OR	Final Multivariable model
	(95% CI)	OR (95% CI)
Gender	1.37 (0.51–3.66)	
Living situation	1.50 (0.44–5.10)	
Age	1.04 (0.98–1.10)	
Age at diagnosis	1.02 (0.973–1.10)	1.09 (1.01–1.18)†
PD duration	0.72 (0.94–1.10)	
No. comorbid conditions	0.88 (0.23–3.39)	
No. of prescription medications	0.34 (0.08–1.59)	
LEDD	1.00 (1.00–1.00)	
LEDD/weight (mg/kg)	1.09 (1.02–1.17)*	1.17 (1.04–1.31)†
UPDRS II	1.08 (1.02–1.14)*	
UPDRS III	1.08 (1.02–1.15)*	1.10 (1.02–1.19)†
H&Y (0–3, less severe) (4 and 5, more severe)	0.19 (0.05–0.77)*	
FOG-Q	1.00 (0.91–1.09)	
ACE-R	0.96 (0.91–1.01)	
MMSE	0.74 (0.58–0.94)*	
STAI	1.02 (0.972–1.06)	0.90 (0.82–0.98)†
BDI	1.11 (1.04 1.19)*	1.23 (1.07–1.41)†
SCOPA-AUT	1.03 (0.97–1.09)	
SCOPA-AUT gastrointestinal	1.16 (1.01–1.33)*	
SCOPA-AUT urinary	0.99 (0.86–1.14)	
SCOPA-AUT cardiovascular	0.92 (0.62–1.34)	
SCOPA-AUT thermoregulatory	1.11 (0.88–1.40)	
SCOPA-AUT pupillomotor	0.72 (0.37–1.39)	
SCOPA-AUT sexual function	1.09 (0.87–1.36)	
MCAS	1.15 (0.96–1.38)	

* Significant independent predictors of nutritional status (*p*<.05) in the unadjusted univariable analysis.

† Significant predictors of nutritional status (*p*<.05) in the multivariable analysis.

Abbreviations: ACE-R = Addenbrooke's Cognitive Examination-Revised; BDI = Beck's Depression Inventory; FOG-Q = Freezing of Gait Questionnaire; H&Y = Hoehn & Yahr; LEDD = levodopa equivalent daily doses; MCAS = Modified Constipation Assessment Scale; MMSE = Mini-Mental State Examination; PD = Parkinson's disease; SCOPA-AUT = Scales for Outcomes in Parkinson's disease – Autonomic; SGA = Subjective Global Assessment; STAI = Spielberger Trait Anxiety Inventory; UPDRS = Unified Parkinson's Disease Rating Scale.

Regression modelling, used to identify the variables associated with log-adjusted PG-SGA score, revealed that living situation, BDI score and UPDRS III score were significant predictors in the final model (Table 4) with more depressive symptoms and more severe motor symptoms predictive of poorer nutritional status. The overall model fit was R2 = 0.31. UPDRS III and BDI were therefore predictive of both SGA category and PG-SGA score.

**Table 4:** Significant predictors of log-adjusted PG-SGA score.

	Coefficient	95% Confidence Interval	p value
Intercept	0.16	0.03–0.30	.014
Living Situation (referent = with others)	0.14	0.01–0.26	.031
BDI score	0.02	0.01–0.02	.000
UPDRS III score	0.01	0.01–0.02	.000

Abbreviations: BDI = Beck's Depression Inventory; PG-SGA = Patient Generated Subjective Global Assessment; UPDRS = Unified Parkinson's Disease Rating Scale.

## Discussion

This is the largest study to characterise PWP diagnosed with malnutrition using a nutrition assessment tool, and it is the first to examine a comprehensive set of potential predictors of nutritional status, including many PD-specific factors.

The combination of a number of these factors may result in poor intake and therefore greater nutritional risk. The nutrition impact section of the PG-SGA score represented the greatest differences between the well-nourished and malnourished in the PG-SGA assessment [31], and a number of the symptoms captured, including nausea, lack of appetite, dysphagia, sensory changes and constipation are symptoms associated with PD [12], [13]. Therefore, these symptoms are important influencers of decreased food intake and malnutrition in PD.

These symptoms may have a greater impact on intake as a result of living situation. Overcoming a lack of motivation to eat may be extremely difficult when there is no one else in the household to encourage eating.

Depression in PD may contribute to this lack of motivation to eat, and as in other groups [1], an increase in depressive symptoms was related to poorer nutritional status in the current sample [14]. Improvements in depression can result in improved body weight [35]. Conversely, improvements in nutritional status can have a positive effect on depression due to improved nutrient intake [36].

Despite the well-documented positive relationship between depression and anxiety in PWP [37], an increase in depressive symptoms but a decrease in anxiety were associated with increased nutrition risk. There has been very limited research regarding the role of anxiety in malnutrition perhaps due to the large degree of overlap in the symptoms of depression and anxiety.

Depression and disease severity both emerged as important factors in all of the relationships explored in the current study which is not surprising considering that a significant relationship between the two has previously been reported [37]. Greater disability in daily tasks such as shopping, cooking and eating resulting from more severe disease may also exacerbate the effect of the nutrition impact symptoms and living situation on intake.

Disease severity is related to the age at diagnosis, not age *per se* or absolute disease duration. An older age at PD diagnosis typically results in more rapid motor symptom progression [38], [39]. A person diagnosed at the age of 70 years experiences an increase of one stage of the H&Y scale in half the time of someone diagnosed at the age of 50 years [38]. This may explain the association in the current study between nutritional status and older age at diagnosis but not age or disease duration. Older age is also associated with an accelerated loss of lean body mass [40], and an accelerated deterioration in motor function coupled with a higher likelihood of lean body mass loss might result in acute and rapid effects on nutritional status. These results support previous studies reporting that age was not a significant predictor of malnutrition risk in PD [14] while age at diagnosis was a significant predictor of increased weight loss [41].

At the same time, energy expenditure may be increased by the presence of dyskinesias resulting from increased dopaminergic treatment, particularly adjusted for body weight [21], [42]. In the current study, LEDD (mg/kg) was significantly higher in the malnourished group and also increased the odds of being classified as malnourished. Weight loss may result in an increased risk of developing dyskinesias [43] that may, in turn, exacerbate weight loss and the risk of malnutrition. The relationship between LEDD (mg/kg) and dyskinesias may occur in a dose-response fashion, with the number of participants experiencing dyskinesia increasing from 3% to 29% in the lowest to highest quartiles of levodopa (mg/kg) respectively [43]. It has been suggested that the adjustment of PD medications to weight changes may be one of the most important modifiable risk factors for developing dyskinesias [44], and this may also have positive effects on nutritional status.

There were also a number of factors that could be expected to predict nutritional status which were not supported in the current study. Polypharmacy (>5 medications [3]) is considered a risk factor in the elderly. Both groups reported a median of 5 prescription medications, and 86% of the participants were taking 4 or more prescription medications, not only for their Parkinson's disease but also for the management of other medical conditions. The definition of polypharmacy for prognostic purposes in PWP may require a higher cut-off. Alternatively, it may not be the absolute number of medications but instead the amount of dopaminergic medication that plays a role as previously discussed.

Dementia and poor cognitive function are also typically considered risk factors for poor nutrition. However, neither ACE-R nor MMSE scores were significant predictors of nutritional status or PG-SGA score. This could be due to sampling bias where PWP with cognitive impairment and/or their spouses chose not to participate. Only 4 well-nourished (4%) and 2 malnourished (11%) scored ≤25 points on the MMSE which is similar to results reported by Wang, et al [14] where the average MMSE score was not significantly predictive of nutritional status. Although the presence of dementia according to the ACE-R (≤83 points) was higher in each group (27% well-nourished, 47% malnourished), this also did not influence the nutritional status of the current sample.

While the total SCOPA-AUT score was not significantly different between the groups, it was higher in the malnourished group. A significant contributor to that difference was the gastrointestinal sub-score. This was reflected in a higher MCAS score in the malnourished group indicating more bowel symptoms and resulting in a lower self-rated satisfaction with bowel function. The greatest number of nutrition impact symptoms on the PG-SGA in this same sample were constipation (60%), early satiety (53%), diarrhoea (50%) and loss of appetite (40%) [31]. Perhaps the SCOPA-AUT is not the best tool by which to measure the severity of gastrointestinal symptoms [45] and future studies could include more objective measures of gastrointestinal dysfunction.

PG-SGA Worksheets 2 (disease conditions) and 3 (metabolic stress) did not contribute to the overall PG-SGA score in this sample. Therefore, the triage score for PD may need to be lower than that in acute diseases, such as cancer and chronic kidney disease. This should be explored further in future research. The analysis resulted in motor symptom severity and depressive symptoms as significant predictors for both SGA and PG-SGA score. The PG-SGA could potentially be complemented by motor symptom and depression assessments to determine risk of malnutrition in PD.

### Strengths

The strengths of this study include its sample size and inclusion of a wide range of ages and disease durations. In addition, a number of potential predictors of nutritional status were explored with a diagnosis of malnutrition using a nutrition assessment tool. Previous studies have focused on BMI or weight loss, and there is already some understanding of factors predicting lower BMIs or significant amounts of weight loss, but not for malnutrition *per se*.

### Limitations

Limitations include the fact that the sampling failed to access the PWP at the more severe end of the disease (H&Y 4, 5). However, these PWP may already reside in aged care facilities and therefore are not within the community-dwelling population. Given that the majority of participants fell within the H&Y 2–3 categories, the application of the current results to PWP in the other H&Y categorisations should occur with caution.

It is recognised that the risk of malnutrition increases among patients/residents of acute and aged care facilities [24], [46]. Therefore hospitalisation or placement into a long-term facility, in its own right, could place someone at nutrition risk. The exclusion of acute care patients and residents of aged care facilities may under-report the issue in the wider PD population particularly as PWP in aged care settings tend to have greater disability and dependency [47]. The aim of this study, however, was to characterise the issue of malnutrition within community-dwelling adults living in their own homes. Documenting the issue may provide the impetus to incorporate regular nutrition screening processes into community-based health care settings in order to identify those in need of further assessment and intervention.

In addition, due to the geographic limitations of the study, the majority of the participants lived in the urban area of Brisbane, Queensland and therefore the study did not take into account potential geographical inequalities in nutritional status. People living in rural areas may not have similar access to services and food supplies as urban-dwellers.

Finally, due to the cross-sectional nature of the study, causality cannot be determined. Many of the factors explored, such as depression, LEDD/weight and more severe disease status, could be exacerbated by malnutrition or contribute to malnutrition. Future longitudinal studies should be conducted to help determine precedence. And, while a number of factors were included in the current analysis, the overall model fit for the PG-SGA score was weak. Additional research could explore in more detail the specific items (eg non-motor symptoms such as sense of smell and taste, presence of inflammation and inflammatory cytokines) that contribute to poor nutritional status.

### Recommendations

The relationship between disease severity, depression and nutritional status warrants a multidisciplinary approach to PD management including neurologists, psychologists and nutrition professionals. The effective management of PD symptoms may have a positive effect on nutritional status, and nutritional status should not be overlooked in the management of PD symptoms. Nutrition screening processes should be established in PWP, particularly in those with more severe disease and depressive symptoms. Health professionals should provide referrals to community-based services and Parkinson's disease support groups to assist with food-related tasks such shopping and cooking, to provide the social aspect of eating and to assist with mood. Review of dopaminergic medication levels should be reviewed with changes in body weight to prevent unnecessary dyskinesias and nutritional status decline.

## Conclusion

Parkinson's disease and its management result in a number of potential risk factors for malnutrition. The malnourished in this study scored poorer on the majority of the assessments than did the well-nourished. More severe motor symptoms and more depressive symptoms were predictive of both malnutrition (SGA-B) and a higher PG-SGA score. Other factors that contributed to one or the other were an older age at diagnosis, higher LEDD/weight and living alone. While nutrition screening should occur for all PWP, it may be particularly important for the older patients at diagnosis, those living alone and those who are depressed.
